# Rates of Primary Care and Integrated Mental Health Telemedicine Visits Between Rural and Urban Veterans Affairs Beneficiaries Before and After the Onset of the COVID-19 Pandemic

**DOI:** 10.1001/jamanetworkopen.2023.1864

**Published:** 2023-03-07

**Authors:** Lucinda B. Leung, Caroline Yoo, Karen Chu, Amy O’Shea, Nicholas J. Jackson, Leonie Heyworth, Claudia Der-Martirosian

**Affiliations:** 1 Center for the Study of Healthcare Innovation, Implementation & Policy, Veterans Affairs Greater Los Angeles Healthcare System, Los Angeles, California; 2 Division of General Internal Medicine–Health Services Research, David Geffen School of Medicine, University of California Los Angeles, Los Angeles; 3 Veterans Emergency Management Evaluation Center, Department of Veterans Affairs, North Hills, California; 4 Center for Comprehensive Access & Delivery Research and Evaluation, Iowa City Veterans Affairs Health Care System, Iowa City; 5 Department of Internal Medicine, University of Iowa Carver College of Medicine, Iowa City; 6 Office of Connected Care/Telehealth Services, Veterans Health Administration, Washington, DC; 7 Department of Medicine, University of California San Diego School of Medicine, San Diego

## Abstract

**Importance:**

Telemedicine can increase access to care, but uptake has been low among people living in rural areas. The Veterans Health Administration initially encouraged telemedicine uptake in rural areas, but telemedicine expansion efforts have broadened since the COVID-19 pandemic.

**Objective:**

To examine changes over time in rural-urban differences in telemedicine use for primary care and for mental health integration services among Veterans Affairs (VA) beneficiaries.

**Design, Setting, and Participants:**

This cohort study examined 63.5 million primary care and 3.6 million mental health integration visits across 138 VA health care systems nationally from March 16, 2019, to December 15, 2021. Statistical analysis took place from December 2021 to January 2023.

**Exposures:**

Health care systems with most clinic locations designated as rural.

**Main Outcomes and Measures:**

For every system, monthly visit counts for primary care and mental health integration specialties were aggregated from 12 months before to 21 months after pandemic onset. Visits were categorized as in person or telemedicine, including video. A difference-in-difference approach was used to examine associations in visit modality by health care system rurality and pandemic onset. Regression models also adjusted for health care system size as well as relevant patient characteristics (eg, demographic characteristics, comorbidities, broadband internet access, and tablet access).

**Results:**

The study included 63 541 577 primary care visits (6 313 349 unique patients) and 3 621 653 mental health integration visits (972 578 unique patients) (6 329 124 unique patients among the cohort; mean [SD] age, 61.4 [17.1] years; 5 730 747 men [90.5%]; 1 091 241 non-Hispanic Black patients [17.2%]; and 4 198 777 non-Hispanic White patients [66.3%]). In fully adjusted models for primary care services before the pandemic, rural VA health care systems had higher proportions of telemedicine use than urban ones (34% [95% CI, 30%-38%] vs 29% [95% CI, 27%-32%]) but lower proportions of telemedicine use than urban health care systems after pandemic onset (55% [95% CI, 50%-59%] vs 60% [95% CI, 58%-62%]), signifying a 36% reduction in the odds of telemedicine use (odds ratio [OR], 0.64; 95% CI, 0.54-0.76). The rural-urban telemedicine gap was even larger for mental health integration (OR, 0.49; 95% CI, 0.35-0.67) than for primary care services. Few video visits occurred across rural and urban health care systems (unadjusted percentages: before the pandemic, 2% vs 1%; after the pandemic, 4% vs 8%). Nonetheless, there were rural-urban divides for video visits in both primary care (OR, 0.28; 95% CI, 0.19-0.40) and mental health integration services (OR, 0.34; 95% CI, 0.21-0.56).

**Conclusions and Relevance:**

This study suggests that, despite initial telemedicine gains at rural VA health care sites, the pandemic was associated with an increase in the rural-urban telemedicine divide across the VA health care system. To ensure equitable access to care, the VA health care system’s coordinated telemedicine response may benefit from addressing rural disparities in structural capacity (eg, internet bandwidth) and from tailoring technology to encourage adoption among rural users.

## Key Points

**Question:**

Are there differential rates of telemedicine use for primary care visits and mental health integration services between rural and urban Veterans Affairs beneficiaries before and after the onset of the COVID-19 pandemic?

**Findings:**

This cohort study included 63.5 million primary care and 3.6 million mental health integration visits across 138 Veterans Affairs health care systems nationally from March 16, 2019, to December 15, 2021. Despite initial increases in telemedicine use among rural health care systems, rural-urban telemedicine disparities in primary care services and mental health integration services increased during the pandemic.

**Meaning:**

This study suggests that, despite a coordinated federal response, there is a rural-urban digital divide after the onset of the COVID-19 pandemic, particularly in mental health integration services.

## Teaser

This cohort study examines rural-urban differences in telemedicine use for primary care and for mental health integration services among Veterans Affairs (VA) beneficiaries before and after the onset of the COVID-19 pandemic.

## Introduction

Telemedicine can effectively connect patients to clinicians across distance and time, but for several reasons (eg, lack of high-speed internet), uptake of telemedicine has been low among people in rural areas.^1,2^ Telemedicine was initially promoted to close access gaps to primary care, mental health, and other essential health care services^3,4^ among rural populations.^5^ Before the COVID-19 pandemic, the Centers for Medicare & Medicaid Services targeted telemedicine reimbursement only for patients located in rural counties or identified health professional shortage areas.^6^ Although many programs have successfully implemented telemedicine among rural populations,^7,8^ uptake remains challenging because US individuals living in rural areas had less access to digital technologies than those living in urban areas (80% vs 89%) and were less likely to have a broadband internet connection at home (72% vs 77%).^9,10^

The Veterans Health Administration (VHA) serves nearly 5 million rural patients across 50 states and has championed telemedicine to expand health care access, including video visits, telephone care, and secure messaging.^11,12^ Since 2016, the VHA has distributed more than 100 000 tablets for video visits to patients in need.^13^ In 2018, Congress passed the Maintaining Internal Systems and Strengthening Integrated Outside Networks (MISSION) Act and legalized cross-state telemedicine delivery.^14^ In 2019, a national VHA initiative created 18 regional hubs to provide telemedicine visits for understaffed clinics.^15^ Although the Veterans Affairs (VA) telemedicine infrastructure had been solidly in place for years, telemedicine use markedly increased in the VA health care system^12^ and other health care systems^16^ when the COVID-19 pandemic began.

This public health emergency affords us an opportunity to close evidence gaps about telemedicine delivery within primary care settings.^17^ Veterans Affairs primary care teams include primary care clinicians, as well as integrated mental health specialists, to comprehensively care for both physical and mental health needs of the entire patient population.^18^ Given long-standing mental health access gaps among rural populations, there is also a specific need to explore telemedicine delivery of mental health integration services, which are delivered directly in the primary care setting by integrated specialists (eg, nurse care managers, embedded psychologists) based on effective collaborative care models.^1^ It is possible that initial telemedicine investments in rural areas conferred an advantage to rural VA health care sites and therefore led to higher rates of telemedicine use during the pandemic. Alternatively, widespread telemedicine expansion, without addressing underlying reasons for digital inequity among rural sites, may result in telemedicine uptake among rural areas lagging behind uptake among urban areas. That is, early telemedicine investments in rural areas may have been outpaced by broader pandemic-related initiatives to expand telemedicine use across all VA health care systems nationally. Our study objective was to investigate rural-urban differences in telemedicine use for primary care and mental health integration services across all VA health care systems nationally before and after the onset of the COVID-19 pandemic.

## Methods

### Study Design

This was a difference-in-difference cohort study using a retrospective observational cohort of VA primary care and mental health integration outpatient visits from March 16, 2019, to December 15, 2021, which represented 1 year after the COVID-19 vaccine became available. This study followed the Strengthening the Reporting of Observational Studies in Epidemiology (STROBE) reporting guideline. The study was part of an ongoing VA quality improvement effort approved by the VA Greater Los Angeles Healthcare System institutional review board as non–human participants research and, therefore, exempt from informed consent requirements.

### Data Sources

Patient-level demographic and clinical characteristics, as well as the date and modality of outpatient visits, were obtained from the Corporate Data Warehouse. Using VA order entry data for prosthetics and devices, we obtained tablet distribution data. To identify the census block of each veteran’s home address, we spatially merged latitude and longitude data with the 2020 US Census Bureau TIGER (Topologically Integrated Geographic Encoding and Referencing)/Line shapefile, which contained geographic entity codes. For each census block, the number of fixed broadband internet providers offering broadband download speeds of 25 megabits per second (MB/s) or more and upload speed of 3 MB/s or more was identified from the December 2020 Federal Communications Commission Form 477.^19^

### Patient Population

Our study cohort included all veterans with an outpatient primary care or mental health integration visit during the study period. We examined all in-person, telephone, and video visits, as well as interactions via secure message. Available data were aggregated across all VA health care systems (n = 138); each system represents 1 VA hospital and its surrounding affiliated community-based clinics.

### Outcomes

Given differential rates of baseline telemedicine use,^20^ primary care visits and mental health integration specialty visits were assessed independently (see eTable 1 in Supplement 1 for clinic code definitions). Encounters were classified into 4 mutually exclusive categories: in person, telephone, video based (including video to home and video to clinic), or secure message. Once encounters were categorized, we assessed the monthly number of completed visits occurring in person, via telemedicine (ie, telephone, video, and secure message), or as a video visit. Visits were aggregated to the health care system level and reported per capita (per 1000 patients). Video visits are examined separately because this modality is the most highly reimbursed across payors. Finally, visits were categorized as being before the onset of the pandemic (March 16, 2019, to March 15, 2020) or after the onset of the pandemic (March 16, 2020, to December 15, 2021).

### Exposure: Rural vs Urban VA Health Care System

We followed Rural-Urban Commuting Area^21^ classifications developed by the Department of Agriculture and the Department of Health and Human Services. A health care system with most locations designated as rural was categorized as rural.

### Covariates

Patient demographic characteristics associated with telemedicine use were aggregated at the health care system; these included age, sex, case mix via the Charlson Comorbidity Index, and self-reported race and ethnicity. We reported the percentage of the patient population that was Black or African American (other race categories included American Indian, Alaska Native, Asian, multiracial, Pacific Islander, and White) and that was Hispanic (other ethnicity categories included not Hispanic, missing, more than 1 ethnicity, and unknown). Racial and ethnic minority groups have been found to use telemedicine differently than White patients.^22,23,24,25,26^ Broadband availability at download speeds of 25 MB/s or more and upload speed of 3 MB/s or more was evaluated for each patient based on their residential census block,^19^ as was having a VA-issued tablet (from VA distribution records).^13,27^ Within each health care system, we also determined the number of unique patients with primary care and mental health integration encounters.

### Statistical Analysis

We analyzed data from December 2021 to January 2023. This study focused on health care system adoption and telemedicine implementation, including video-based services, and examined organizational factors aggregated to VA health care systems.^28^ We characterized 138 health care systems by site-level covariates associated with the expansion and use of telemedicine services.^26,28,29,30,31,32^ These system-level characteristics were described before vs after the onset of the pandemic with numeric data reported using means and SDs and categorical data using percentages. Unadjusted comparisons were conducted using the 2-sample independent *t* test or the Wilcoxon rank sum test as appropriate. Characteristics were similarly reported by health care system rurality.

Primary care visits and mental health integration visits were summarized before and after the onset of the COVID-19 pandemic and stratified by rural vs urban VA health care system. Mean (SD) values were reported by visit modality.

In this study, a difference-in-difference approach was used to examine if the urban-rural gap changed during the course of the pandemic (ie, were prepandemic differences similar to pandemic era differences). Generalized linear models with a logit link and binomial distribution estimated the system-level aggregated visit counts, with the total visit counts as the binomial denominator and adjusting for system-level covariates, before and after the onset of the pandemic stratified by VA health care system rurality. Separate models for primary care and mental health integration were assessed. Using this model structure, we also tested the main effects for, and the interaction between, prepandemic vs postpandemic onset and rurality. Odds ratios (ORs) with 95% CIs and predicted probabilities (and delta-method–derived SEs), holding all covariates at their means, were reported.

Our sensitivity analyses excluded secure messages. In addition, we used a multivariable model under a negative binomial setting to estimate the total number of visits for each VA health care system before and after the onset of the pandemic stratified by rurality. For all analyses, significance was determined using a 2-tailed type I error rate of .05. Data were analyzed in Stata statistical software, version 17.0 (StataCorp LLC).

## Results

We conducted an observational study of 63 541 577 primary care visits (6 313 349 unique patients) and 3 621 653 mental health integration visits (972 578 unique patients) from 138 VA health care systems nationwide. Among the full study cohort, there were 6 329 124 unique patients (mean [SD] age, 61.4 [17.1] years; 5 730 747 men [90.5%]; 1 091 241 non-Hispanic Black patients [17.2%]; and 4 198 777] non-Hispanic White patients [66.3%]) from 45 rural (including highly rural and insular islands) and 93 urban VA health care systems. Compared with primary care visits, a higher proportion of mental health integration visits were telemedicine. Overall, all primary care visits consisted of 31 762 268 telemedicine visits [50.0%], including 2 595 052 video visits [4.1%] and 31 779 309 in-person visits [50.0%], whereas mental health integration visits were 2 251 882 telemedicine visits [62.2%], including 801 393 video visits [22.1%] and 1 369 771 in-person visits [37.8%].

Table 1 shows the aggregated characteristics of all 138 VA health care systems before and after the onset of the pandemic. After the onset of the pandemic, most system-level characteristics remained similar except for slight decreases in mean (SD) age (from 63.4 [2.8] years to 61.6 [2.9] years; *P* < .001) and median percentage of men (from 92.2% [IQR, 90.2%-93.4%] to 91.7% [IQR, 89.7%-92.9%]; *P* = .02), as well as an increase in mean (SD) Charlson Comorbidity Index (from 1.4 [0.2] to 1.5 [0.2]; *P* < .001). A median 90.4% (IQR, 86.5%-93.5%) of patients lived in a location where 1 or more internet service providers reported available broadband internet access by midstudy, December 2020. Across all study years, a median 1.7% (IQR, 0.9%-3.2%) of patients received VA-issued tablets. When comparing VA health care systems by rurality, there were expected differences in patient age, sex, race and ethnicity, volume of patients per month, and broadband access; however, no rural-urban differences were noted in Charlson Comorbidity Index or tablet distribution.

**Table 1.** VA Health Care System Characteristics, by Rurality and the COVID-19 Pandemic Onset

Characteristic	By rurality		*P* value^a^	By pandemic onset		*P* value^a^
	Urban (n = 93)	Rural (n = 45)		Prepandemic (n = 138)	Postpandemic (n = 138)	
No. of patients per month, mean (SD)	11 665 (6001)	6555 (2323)	<.001	9743 (5433)	10 145 (5770)	.55
Charlson Comorbidity Index, mean (SD)	1.4 (0.2)	1.4 (0.2)	.84	1.4 (0.2)	1.5 (0.2)	<.001
Age, mean (SD), y	61.1 (3.0)	63.1 (2.3)	<.001	63.4 (2.8)	61.6 (2.9)	<.001
Black patients, median (IQR), %	14.7 (8.6-30.8)	3.8 (1.4-6.8)	<.001	9.8 (3.6-21.0)	10.2 (3.8-21.0)	.87
Hispanic patients, median (IQR), %	3.3 (1.9-7.5)	1.5 (0.9-3.5)	<.001	2.4 (1.3-5.9)	2.6 (1.5-6.1)	.61
Men, median (IQR), %	91.0 (88.9-92.6)	92.7 (91.6-93.6)	<.001	92.2 (90.2-93.4)	91.7 (89.7-92.9)	.02
Women, median (IQR), %	9.0 (7.4-11.1)	7.3 (6.4-8.5)	<.001	7.8 (6.6-9.8)	8.3 (7.1-10.3)	.02
Patients with VA-issued tablets, median (IQR), %^b^	1.9 (0.9-3.3)	1.1 (0.8-2.4)	.14	NA	1.7 (0.9-3.2)	NA
Quartiles: patients with VA-issued tablets, No. (%)^b^						
First (0.1%-0.7%)	21 (22.6)	13 (28.9)	.24	NA	34 (24.6)	NA
Second (0.8%-1.6%)	20 (21.5)	15 (33.3)		NA	35 (25.4)	
Third (1.7%-3.1%)	27 (29.0)	8 (17.8)		NA	35 (25.4)	
Fourth (3.2%-9.6%)	25 (26.9)	9 (20.0)		NA	34 (24.6)	
Patients with broadband access, median (IQR), %^c^	91.9 (88.6-94.5)	86.5 (84.9-90.4)	<.001	NA	90.4 (86.5-93.5)	NA
Quartiles: patients with broadband access with download speeds ≥25 MB/s and upload speeds ≥3 MB/s, No. (%)^c^						
First (35.7%-86.4%)	12 (12.9)	22 (48.9)	<.001	NA	34 (24.6)	NA
Second (86.5%-90.3%)	24 (25.8)	11 (24.4)		NA	35 (25.4)	
Third (90.4%-93.4%)	28 (30.1)	7 (15.6)		NA	35 (25.4)	
Fourth (93.5%-96.2%)	29 (31.2)	5 (11.1)		NA	34 (24.6)	

Abbreviations: MB, megabits; NA, not applicable; VA, Veterans Affairs.

^a^ *t* Tests or Wilcoxon rank sum tests were conducted for all comparisons by rurality and pandemic onset.

^b^ VA-issued tablets requested and distributed from March 2019 through December 2021.

^c^ Broadband internet availability data by the Federal Communications Commission (December 2020).

Unadjusted analyses showed that mean (SD) monthly primary care visits per 1000 patients increased after the onset of the pandemic among rural VA health care systems (from 1232 [9] to 1256 [26]) as well as urban VA health care systems (from 1237 [12] to 1267 [24]) (Table 2). For mental health integration visits, however, mean (SD) monthly visits per 1000 patients decreased among urban health care systems (from 1323 [25] to 1315 [19]) and rural systems (from 1342 [24] to 1320 [21]). In total, however, mean monthly visits for both specialties increased across rural and urban systems. Telemedicine care patterns were similar between rural and urban VA health care systems 12 months prior to the onset of the pandemic (Figure). Observed rural-urban differences in telemedicine use remained constant 21 months after the onset of the pandemic.

**Table 2.** Unadjusted Mean Monthly Visits per 1000 Patients for Primary Care and Mental Health Integration Specialties, by VA Health Care System Rurality and Pandemic Onset

Visit type	Visits, mean (SD), No.			
	Urban health care systems		Rural health care systems	
	Prepandemic onset	Postpandemic onset	Prepandemic onset	Postpandemic onset
Primary care				
In-person visits	863 (28)	487 (90)	817 (30)	565 (97)
Telemedicine visits^a^	373 (20)	780 (112)	416 (28)	691 (120)
Video visits	12 (1)	79 (11)	25 (2)	47 (6)
Telephone visits	345 (19)	674 (109)	380 (27)	629 (116)
Secure messages	17 (1)	27 (2)	11 (1)	16 (2)
Total visits	1237 (12)	1267 (24)	1232 (9)	1256 (26)
Mental health integration				
In-person visits	989 (34)	197 (41)	959 (21)	305 (60)
Telemedicine visits^a^	334 (18)	1118 (49)	383 (10)	1015 (67)
Video visits	71 (10)	443 (37)	113 (8)	287 (24)
Telephone visits	263 (8)	673 (63)	269 (11)	728 (63)
Secure messages	0.4 (0.1)	1 (0.3)	0.2 (0.2)	0.3 (0.2)
Total visits	1323 (25)	1315 (19)	1342 (24)	1320 (21)
Total				
In-person	889 (28)	478 (88)	836 (29)	562 (95)
Telemedicine^a^	379 (19)	808 (110)	421 (28)	711 (119)
Video	15 (1)	100 (12)	29 (2)	56 (7)
Telephone visits	348 (18)	682 (108)	381 (26)	639 (115)
Secure messages	16 (1)	26 (2)	11 (1)	15 (2)
Total visits	1268 (13)	1286 (26)	1257 (10)	1272 (27)

Abbreviation: VA, Veterans Affairs.

^a^ Telemedicine visits include telephone, video, and secure messaging.

**Figure.**
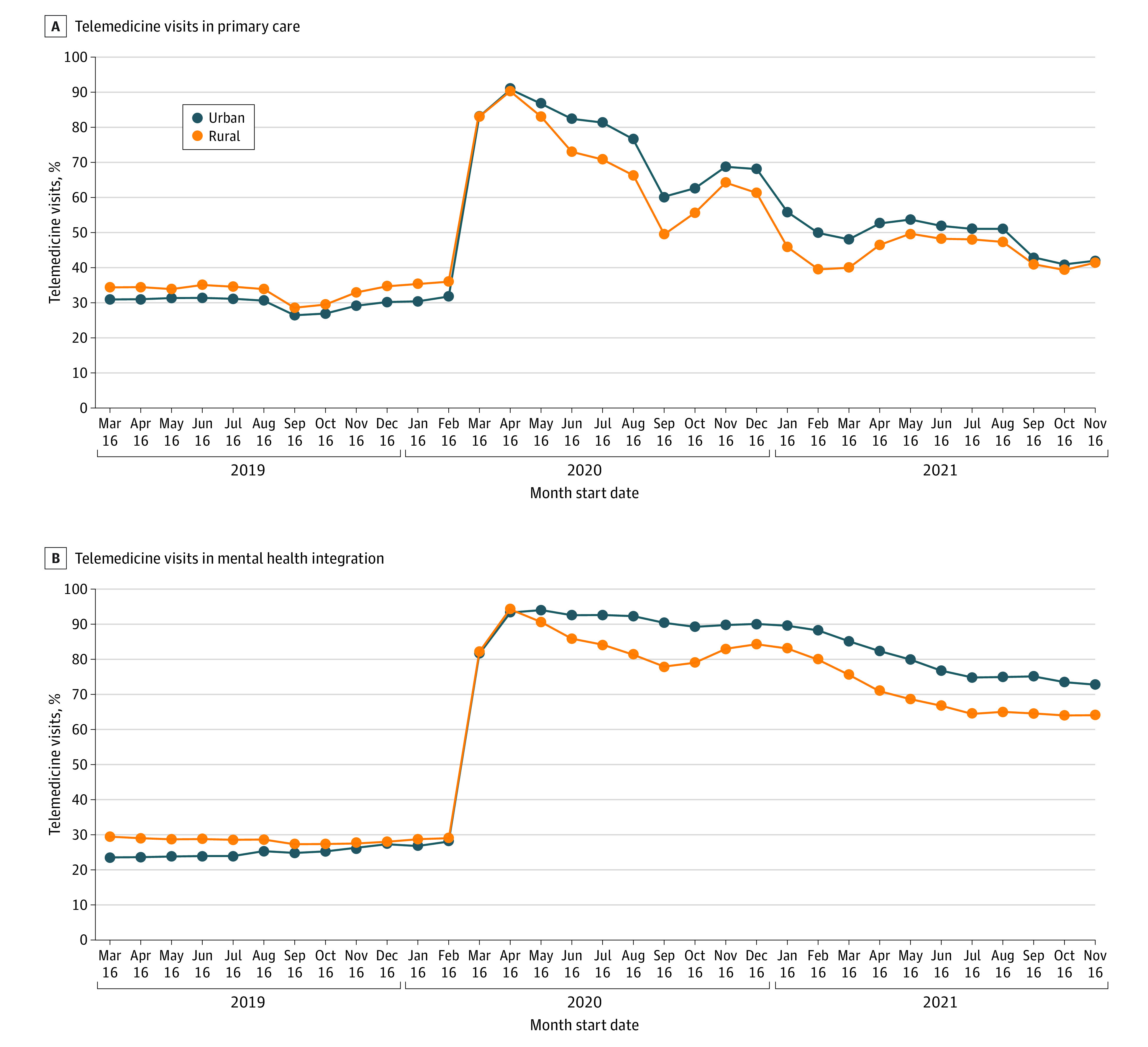
Unadjusted Mean of Monthly Telemedicine and Video Visits for Primary Care and Mental Health Integration Specialties, by Veterans Affairs Health Care System Rurality Over the Study Period

Correspondingly, for primary care services, rural VA health care systems initially had greater telemedicine use than urban systems (34% vs 30%), which switched after the onset of the pandemic (55% vs 61%) (eTable 2 in Supplement 1). For mental health integration services, rural systems also had greater telemedicine use than urban systems (29% vs 25%), which also switched after the onset of the pandemic (76% vs 84%). Few video visits occurred across rural and urban health care systems (before the pandemic, 2% vs 1%; after the pandemic, 4% vs 8%).

Adjusted regression analyses demonstrated similar findings (Table 3). For primary care services, rural VA health care systems initially had greater telemedicine use than urban systems (34% [95% CI, 30%-38%] vs 29% [95% CI, 27%-32%]), which switched after the onset of the pandemic (55% [95% CI, 50%-59%] vs 60% [95% CI, 58%-62%]) (eFigure in Supplement 1), for a 36% reduction in the difference in the odds of telemedicine use after the pandemic (OR, 0.64; 95% CI, 0.54-0.76; *P* < .001) (eTable 3 in Supplement 1). In comparison, the rural-urban telemedicine gap grew even larger for mental health integration services (OR, 0.49; 95% CI, 0.35-0.67; *P* < .001), with less rural than urban telemedicine use after the pandemic (rural: before the pandemic, 29% [95% CI, 23%-36%] vs after the pandemic, 77% [95% CI, 72%-82%]; urban: before the pandemic, 25% [95% CI, 22%-29%] vs after the pandemic, 85% [95% CI, 82%-87%]).

**Table 3.** Adjusted Percentages by Visit Modality for Primary Care and Mental Health Integration Specialties, by VA Health Care System Rurality and Pandemic Onset^a^

Visit type	Health care system rurality	% (95% CI)		
		Telemedicine visits^b^	Video visits	In-person visits
Primary care				
Prepandemic onset	Urban	29 (27-32)	1 (1-1)	71 (68-73)
	Rural	34 (30-38)	2 (2-3)	66 (62-70)
Postpandemic onset	Urban	60 (58-62)	6 (5-6)	40 (38-42)
	Rural	55 (50-59)	5 (4-6)	45 (41-50)
Difference: prepandemic vs postpandemic, %	Urban	31	5	−31
	Rural	21	3	−21
	Difference	−10	−2	10
Mental health integration				
Prepandemic onset	Urban	25 (22-29)	5 (4-7)	75 (71-78)
	Rural	29 (23-36)	8 (4-12)	71 (64-77)
Postpandemic onset	Urban	85 (82-87)	34 (31-37)	15 (13-18)
	Rural	77 (72-82)	22 (18-27)	23 (18-28)
Difference: prepandemic vs postpandemic, %	Urban	60	29	−60
	Rural	48	14	−48
	Difference	−12	−15	12

Abbreviation: VA, Veterans Affairs.

^a^ Binomial logistic regression models adjusted for aggregated health care system–level characteristics (ie, patient demographic characteristics, number of primary care patients, access to broadband internet, and distributed VA tablets).

^b^ Telemedicine visits include telephone, video, and secure messaging.

For the subset of video visits, there was a rural-urban divide for primary care (OR, 0.28; 95% CI, 0.19-0.40; *P* < .001) and mental health integration services (OR, 0.34; 95% CI, 0.21-0.56; *P* < .001) (eTable 3 in Supplement 1). Use of video visits, a subset of telemedicine visits, was low overall and had even larger rural-urban disparities. Rural health care systems initially had greater video use than urban systems (primary care, 2% [95% CI, 2%-3%] vs 1% [95% CI, 1%-1%]; mental health integration, 8% [95% CI, 4%-12%] vs 5% [95% CI, 4%-7%]), which switched after the onset of the pandemic (primary care, 5% [95% CI, 4%-6%] vs 6% [95% CI, 5%-6%]; mental health integration, 22% [95% CI, 18%-27%] vs 34% [95% CI, 31%-37%]). Among model covariates, VA systems in which patients had the highest access to broadband internet used more video visits (but not telemedicine visits). Veterans Affairs health care systems serving higher proportions of Black patients and distributing fewer VA-issued tablets had reduced odds of using video visits (but not telemedicine visits) for mental health integration services.

Results of sensitivity analyses excluding secure messages were similar to the main results. Additional analyses showed that total visits were similar by rurality and across time periods after adjusting for covariates, suggesting that increased telemedicine visits are substituting for previous in-person services. The interaction between onset of the pandemic and system rurality was significant, however, and represented a reduction in rates of visits in rural vs urban health care systems over time (primary care incidence rate ratio [IRR], 0.98; 95% CI, 0.96-0.99; *P* = .006; mental health integration IRR, 0.97; 95% CI, 0.94-1.0; *P* = .04) (eTables 4 and 5 in Supplement 1).

## Discussion

Despite early increases in telemedicine use in rural areas associated with national telemedicine initiatives targeting rural populations, including widespread tablet provision to patients for video visits,^13^ there was a rural-urban telemedicine divide across the VA health care system since the onset of COVID-19. To our knowledge, this is the first study to examine prepandemic and postpandemic patterns of telemedicine use by rurality across the VA health care system nationally. Telemedicine expansion for rural VA health care systems lagged behind urban ones, possibly because underlying reasons for digital inequity were not addressed. Rural telemedicine disparities have been widely reported among patients and hospitals globally.^33,34,35,36,37,38,39,40,41^ Consistent with prior research, our large national VA health care system sample substantiates concerns among rural health scholars of disparities during the pandemic-driven shift to virtual care.^2,42^ Even with significant VA investment to increase care access (eg, early telemedicine infrastructure, legalization of interstate care) and initiatives designed to bridge the digital divide (eg, as-needed tablet distribution), rural telemedicine disparities have not been remedied. Future telemedicine research and implementation efforts should target intervention at multiple levels to overcome the growing rural-urban digital divide.

Video visits are the most highly reimbursed telemedicine modality,^43^ but they constituted a minority of VA health care services provided to VA patients, who are often older, are of lower socioeconomic status, and have higher medical complexity than veterans treated outside the VA health care system.^44^ Rural VA health care systems delivered even fewer video visits than urban systems, suggesting greater barriers to video visits than other telemedicine modalities for rural populations. Lack of reliable, high-quality broadband internet access,^33^ which our study showed is inversely associated with rurality and use of video health care visits, plays a contributing role. Moreover, reliable internet access has been associated with not just telemedicine use but also with satisfaction with telemedicine services,^35^ which likely begets more telemedicine visits. Additional barriers to telemedicine exist at health system (eg, cost, reimbursement), clinician (eg, technical challenges, resistance to change), and patient (eg, age, education) levels and likely require multipronged interventions to overcome.^37^ Although the VA health care system has attempted to dismantle health system barriers, it continues to experience clinician- and patient-level barriers in telemedicine implementation. We did not observe an association between VA health care systems’ distribution of tablets and patients’ broader use of video visits for primary care or integrated mental health visits. Tablets have been disproportionately used for mental health specialty visits,^13^ which may account for this finding, however. As part of tablet distribution efforts, the VA has engaged in and will likely need to continue outreach to improve veterans’ digital literacy and to increase video use among tablet users. Consistent with prior studies,^22,23^ we also noted differences between Black and White veterans in video visit uptake at the health care system level. A recent scoping review highlighted that the most effective and equitable health system telemedicine implementations are intentionally patient centered and culturally tailored.^34^

This study also noted differential patterns of telemedicine use between primary care clinicians and mental health integration specialists and differential service use overall between both specialties. Both specialties serve the same VA primary care population, with mental health integration caring for a smaller subset of patients. Compared with urban health care systems, telemedicine expansion lagged for rural systems, more so for mental health integration than primary care services. Rural VA populations have been reported to have poor access to mental health treatments,^4^ and this study suggests that the pandemic may worsen mental health access gaps, most of which are now delivered virtually.^23^ Mental health care has long been difficult to access for rural Americans, and findings here and elsewhere suggest that access has gotten worse during the pandemic.^45^ Scholars have called attention to the need for telemedicine prioritization in rural areas (eg, directing incentives for proactive telemedicine uptake) and innovation in mental health care access (eg, technology customization for rural populations)^46,47,48^ given co-occurring telemedicine disparities and specialty access disparities. Findings caution widespread unmonitored implementation of telemedicine for mental health given the lower uptake of services among rural than urban health care systems. Because there are likely rural-urban differences in mental health care use irrespective of telemedicine expansion (related to stigma or cultural norms, mental health workforce, or community referral patterns),^49^ solutions will likely need to incorporate increased access to patient-preferred mental health care modalities, whether in-person and/or telemedicine.

### Strengths and Limitations

The strength of this study lies in its large national sample of the entire VA primary care population, but there are potential limitations to consider. First, we aggregated telemedicine visits to rural and urban VA health care systems, which may have misclassified some rural patients (eg, rural patients who drove to urban health care systems for care). However, this study provides insight into potential improvements to care access if policy changes and interventions were to be targeted at the system level, analogous to efforts aiming to save Medicare-designated critical access hospitals. Second, we did not have data on VA patients who received non-VA care in the community, for which many rural patients (more so than urban patients) may opt to have; however, few primary care or mental health services have been found to be delivered outside the VA health care system.^50^ Third, study generalizability may also be limited to health care systems that had early telemedicine infrastructure; however, implications likely apply to most health care systems that have adopted telemedicine care.

## Conclusions

Despite a coordinated federal response, the VA health care system’s experience suggests that increasing telemedicine use may leave rural patients at risk for poor access to care. Future research and implementation efforts for health care systems must address rural-urban structural inequities (eg, internet bandwidth) and consider tailoring technology to encourage adoption among rural users at all levels (patients, clinicians, and health care systems). As health care systems increasingly rely on telemedicine to deliver necessary medical services, rural hospitals may be subject to greater operational challenges, and rural patients may be subject to increasing inequity in access to care compared with their urban counterparts.

## Notes

Supplement 1. **eTable 1.** Clinic Codes and Definition of Clinic Codes for Primary Care, Mental Health Integration, and Telemedicine Visits **eTable 2.** Unadjusted Percent of Telemedicine, Video, In-Person Visits, for Primary Care and for Mental Health Integration Specialties, by VA Healthcare System Rurality and Pandemic Onset **eFigure.** Predicted Probabilities Pre- and Post-COVID-19 Pandemic Onset by VA Healthcare System Rurality **eTable 3.** Odds Ratios and 95% CIs for Telemedicine, Video, and In-Person Visits in Primary Care and Mental Health Integration Services, Respectively, Using Multivariable Binomial Logistic Regression **eTable 4.** Incidence Rate Ratios and 95% CIs for Telemedicine, Video, and In-Person Visits in Primary Care and Mental Health Integration Services, Using Multivariable Negative Binomial Models **eTable 5.** Predicted Number of Visits for Primary Care and Mental Health Integration Specialties, by VA Healthcare System Rurality and Pandemic Onset

Supplement 2.
Data Sharing Statement
