# Supplementary material for: Rates of Primary Care and Integrated Mental Health Telemedicine Visits Between Rural and Urban Veterans Affairs Beneficiaries Before and After the Onset of the COVID-19 Pandemic
Source: JAMA Netw Open. 2023 Mar 7;6(3):e231864. doi: 10.1001/jamanetworkopen.2023.1864 (PMC9993180; doi:10.1001/jamanetworkopen.2023.1864)
Supplement: Supplement 1. — eTable 1. Clinic Codes and Definition of Clinic Codes for Primary Care, Mental Health Integration, and Telemedicine Visits eTable 2. Unadjusted Percent of Telemedicine, Video, In-Person Visits, for Primary Care and for Mental Health Integration Specialties, by VA Healthcare System Rurality and Pandemic Onset eFigure. Predicted Probabilities Pre- and Post-COVID-19 Pandemic Onset by VA Healthcare System Rurality eTable 3. Odds Ratios and 95% CIs for Telemedicine, Video, and In-Person Visits in Primary Care and Mental Health Integration Services, Respectively, Using Multivariable Binomial Logistic Regression eTable 4. Incidence Rate Ratios and 95% CIs for Telemedicine, Video, and In-Person Visits in Primary Care and Mental Health Integration Services, Using Multivariable Negative Binomial Models eTable 5. Predicted Number of Visits for Primary Care and Mental Health Integration Specialties, by VA Healthcare System Rurality and Pandemic Onset [file jamanetwopen-e231864-s001.pdf]

Supplementary Online Content

Leung LB, Yoo C, Chu K, et al. Rates of primary care and integrated mental health telemedicine visits between rural and urban Veterans Affairs beneficiaries before and after the onset of the COVID-19 pandemic. *JAMA Netw Open.* 2023;6(3):e231864. doi:10.1001/jamanetworkopen.2023.1864

eTable 1. Clinic Codes and Definition of Clinic Codes for Primary Care, Mental Health Integration, and Telemedicine Visits
eTable 2. Unadjusted Percent of Telemedicine, Video, In-Person Visits, for Primary Care and for Mental Health Integration Specialties, by VA Healthcare System Rurality and Pandemic Onset
eFigure. Predicted Probabilities Pre- and Post-COVID-19 Pandemic Onset by VA Healthcare System Rurality
eTable 3. Odds Ratios and 95% CIs for Telemedicine, Video, and In-Person Visits in Primary Care and Mental Health Integration Services, Respectively, Using Multivariable Binomial Logistic Regression
eTable 4. Incidence Rate Ratios and 95% CIs for Telemedicine, Video, and In-Person Visits in Primary Care and Mental Health Integration Services, Using Multivariable Negative Binomial Models
eTable 5. Predicted Number of Visits for Primary Care and Mental Health Integration Specialties, by VA Healthcare System Rurality and Pandemic Onset

This supplementary material has been provided by the authors to give readers additional information about their work.

© 2023 Leung LB et al. *JAMA Network Open.*

eTable 1. Clinic Codes and Definition of Clinic Codes for Primary Care, Mental Health Integration, and Telemedicine Visits

Clinic Code	Definition of Clinic Code	Primary Care	Mental Health Integration	Video	Phone	Secure Message
322	Comprehensive Women's Primary Care Clinic	X				
323	Primary Care Medicine	X				
338	Telephone Primary Care	X				
341	Pediatrics	X				
342	Family Practice	X				
348	Primary Care Shared Appointment	X				
350	Geriatric Patient Aligned Care Team	X				
531	Primary Care for Patients with Serious Mental Illness (SMI)	X				
704	Women's Gender-Specific Preventive Care	X				
534	Mental Health Integrated Care - Individual		X			
539	Mental Health Integrated Care - Group		X			
136	Tele-Post Deployment Integrated Care- Patient Site			X		
137	Tele-Post Deployment Integrated Care- Provider Site			X		
179	Real Time Clinical Video Telehealth to Home- Provider Site			X		
440	Tele-Fittings and Adjustments- Provider Site			X		
444	Compensation and Pension (C&P) Exam via Clinical Video Telehealth- Patient Site			X		
445	Compensation and Pension (C&P) Exam via Clinical Video Telehealth- Provider Site			X		
446	Integrated Disability and Evaluation System (IDES) Exam via Clinical Video Telehealth- Patient Site			X		
447	Integrated Disability and Evaluation System (IDES) Exam via Clinical Video Telehealth- Provider Site			X		
490	TeleTransplant Service- Patient Site			X		
491	TeleTransplant Service- Provider Site			X		
644	National Center Real Time Clinical Video Telehealth- Patient Site			X		
645	National Center Real Time Clinical Video Telehealth- Provider Site			X		
648	Real Time Clinical Video Telehealth with Non-VA Medical Center Location- Provider Site			X		
679	National Center Real Time Clinical Video Telehealth to Home- Provider Site			X		

© 2023 Leung LB et al. *JAMA Network Open.*

690	Real Time Clinical Video Telehealth-Patient Site			X		
692	Real Time Clinical Video Telehealth-Provider Site (Same Station)			X		
693	Real Time Clinical Video Telehealth - Provider Site (Not Same Station)			X		
699	Real Time Clinical Video Telehealth- Same Station Services- Provider Site			X		
708	Tele-Smoking Cessation- Provider Site			X		
723	Tele-Opioid Safety Education- Patient Site			X		
724	Tele-Opioid Safety Education- Provider Site			X		
103	Telephone Triage				X	
147	Telephone Ancillary				X	
148	Telephone/Diagnostic				X	
169	Telephone/Chaplain				X	
178	Telephone Home Based Primary Care				X	
181	Telephone/ Dental				X	
182	Telephone Case Management				X	
199	Telephone Polytrauma/Traumatic Brain Injury (TBI)				X	
216	Telephone Rehabilitation (Rehab) & Support				X	
221	Telephone Visual Impairment Service Team (VIST)				X	
224	Telephone Spinal Cord Injury (SCI)				X	
229	Telephone Blind Rehab Program				X	
324	Telephone Medicine				X	
325	Telephone Neurology				X	
326	Telephone Geriatrics				X	
338	Telephone Primary Care				X	
424	Telephone/Surgery				X	
425	Telephone Prosthetics/Orthotics				X	
428	Telephone/Optometry				X	
441	Telephone Anesthesia				X	
527	Telephone Mental Health				X	
528	Telephone/Homeless Chronically Mentally Ill (HCMI)				X	
530	Telephone/Housing and Urban Development - VA Supportive Housing (HUD-VASH)				X	
536	Telephone Mental Health Vocational Assistance				X	
542	Telephone Post Traumatic Stress Disorder				X	
545	Telephone Substance Use Disorder				X	

© 2023 Leung LB et al. *JAMA Network Open.*

546	Telephone Intensive Community Mental Health Recovery Services (ICMHR)				X	
579	Telephone Psycho-Geriatrics				X	
584	Telephone Psychosocial Rehabilitation Recovery Center (PRRC)				X	
597	Telephone/Residential Rehabilitation Treatment Program (RRTP)				X	
611	Telephone Dialysis				X	
801	Telephone Triage in Veterans Integrated Service Network				X	
802	Telephone Triage out of Veterans Integrated Service Network				X	
189	Store & Forward Telehealth from Home-Provider Site					X
646	National Center Store & Forward Telehealth- Patient Site					X
647	National Center Store & Forward Telehealth- Provider Site					X
694	Store & Forward Telehealth- Patient Site					X
695	Store & Forward Telehealth- Provider Site (Same Division/Station)					X
696	Store & Forward Telehealth- Provider Site (Not Same Station)					X
698	Store & Forward Telehealth from Non-VA Medical Center Location- Provider Site					X
718	Diabetic Retinal Screening					X
719	My HealtheVet Secure Messaging					X

© 2023 Leung LB et al. *JAMA Network Open.*

eTable 2. Unadjusted Percent of Telemedicine, Video, In-Person Visits, for Primary Care and for Mental Health Integration Specialties, by VA Healthcare System Rurality and Pandemic Onset

	Any Telemedicine[a] Visit			Any Video Visit			Any In-Person Visit		
	**Pre-Pandemic**	**Post-Pandemic Onset**	**Diff**	**Pre-Pandemic**	**Post-Pandemic Onset**	**Diff**	**Pre-Pandemic**	**Post-Pandemic Onset**	**Diff**
**Primary Care**									
Urban	30%	61%	31%	1%	6%	5%	70%	39%	-31%
Rural	34%	55%	21%	2%	4%	2%	66%	45%	-21%
**Mental Health Integration**									
Urban	25%	84%	59%	5%	33%	28%	75%	16%	-59%
Rural	29%	76%	47%	9%	21%	12%	71%	24%	-47%
**Total**									
Urban	30%	63%	33%	1%	8%	7%	70%	37%	-33%
Rural	33%	56%	23%	2%	4%	3%	67%	44%	-23%

[a]Telemedicine visits include telephone, video, and secure messaging.

© 2023 Leung LB et al. *JAMA Network Open.*

eFigure. Predicted Probabilities Pre- and Post-COVID-19 Pandemic Onset by VA Healthcare System Rurality

Telemedicine Visits (including telephone, video, and secure messaging)

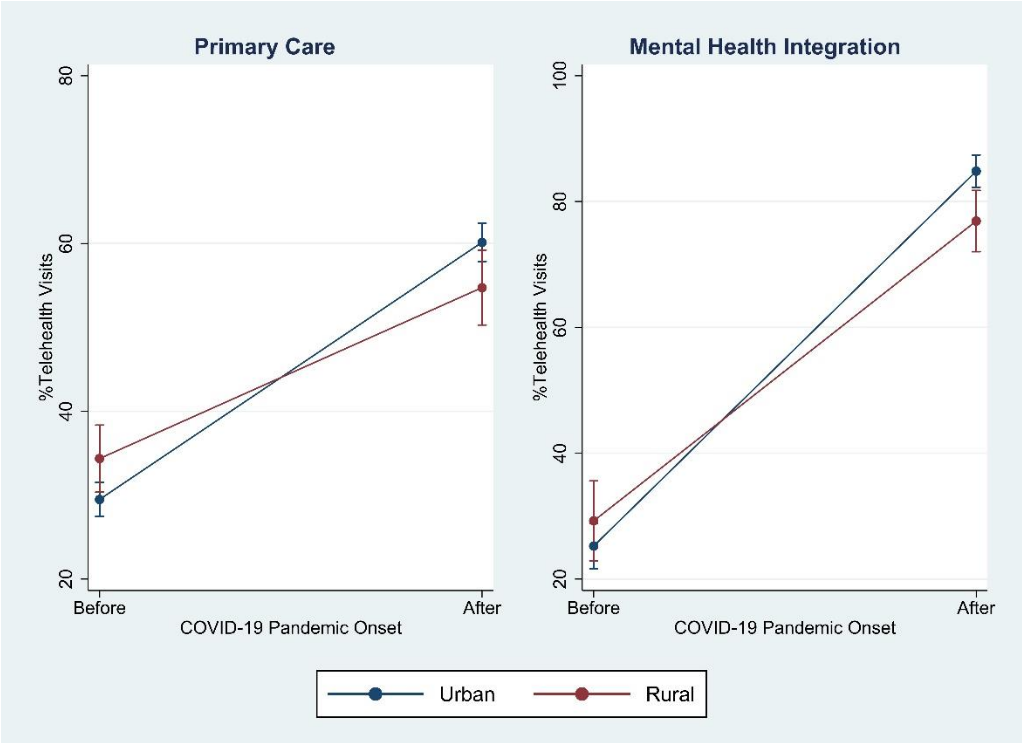

Video Visits

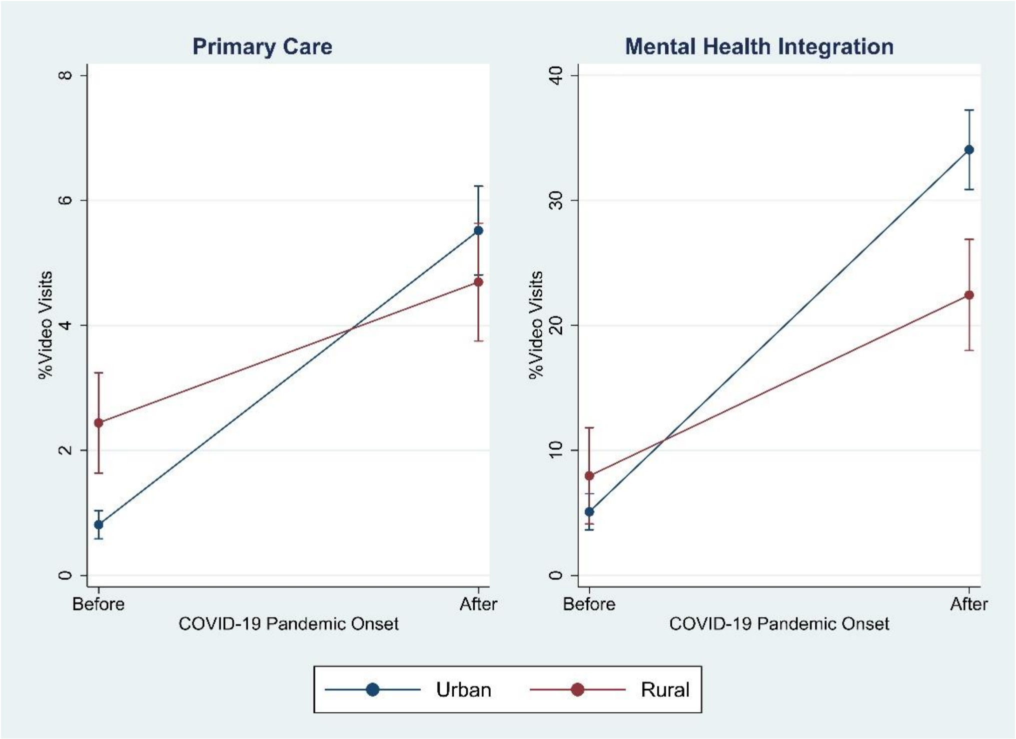

© 2023 Leung LB et al. *JAMA Network Open.*

In-person Visits

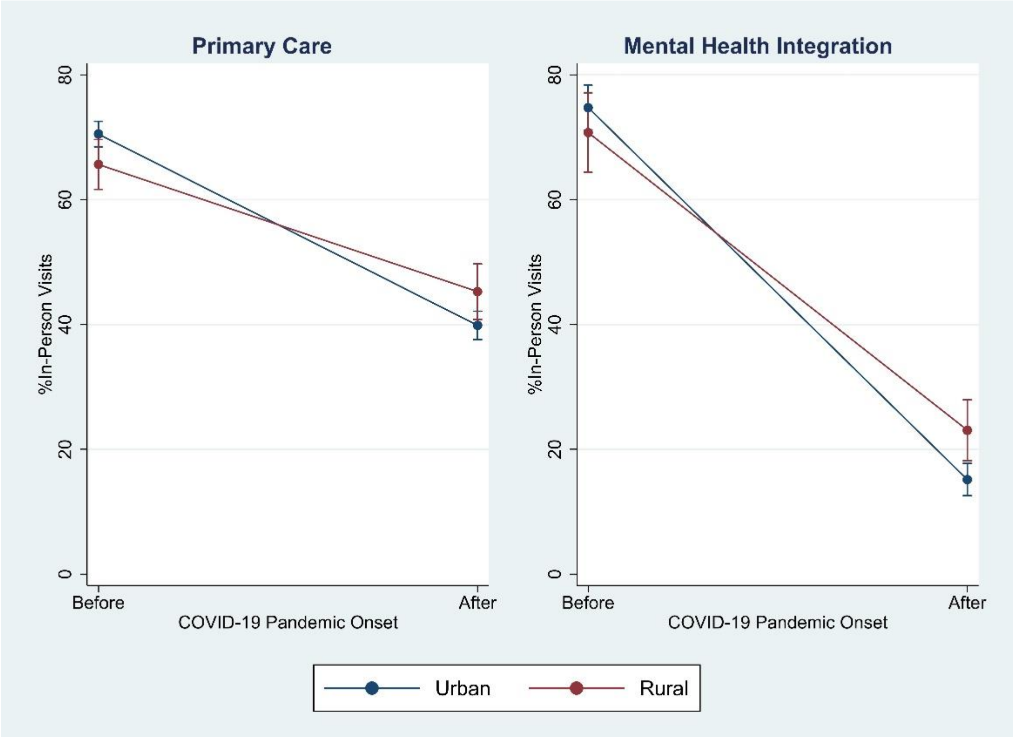

© 2023 Leung LB et al. *JAMA Network Open.*

eTable 3. Odds Ratios and 95% CIs for Telemedicine, Video, and In-Person Visits in Primary Care and Mental Health Integration Services, Respectively, Using Multivariable Binomial Logistic Regression

	**Any Telemedicine Visit[a] OR (95% CI)**	**Any Video Visit OR (95% CI)**	**Any In-Person Visit OR (95% CI)**
**Primary Care**			
Post-Pandemic Onset (versus Pre-)	3.61 (3.27- 4.00)	7.17 (5.44 - 9.43)	0.28 (0.25 - 0.31)
Rural (versus Urban)	1.252 (1.001 - 1.566)	3.06 (1.91 - 4.92)	0.799 (0.639 - 0.999)
Post-Pandemic Onset by Rurality	0.64 (0.54 - 0.76)	0.28 (0.19 - 0.40)	1.56 (1.32 - 1.85)
Mean Charlson Comorbidity Index	1.10 (0.81 - 1.51)	0.73 (0.44 - 1.21)	0.91 (0.69 - 1.11)
Mean Number of Patients per Month	1.000 (1.000 - 1.000)	1.000 (1.000 - 1.000)	1.000 (1.000 - 1.000)
Percent Black Patients	1.002 (0.997 - 1.007)	1.006 (1.001 - 1.014)	0.998 (0.993 - 1.003)
Percent Hispanic Patients	0.996 (0.992 - 0.999)	1.008 (1.001 - 1.015)	1.004 (1.001 - 1.008)
Percent Patients with VA-issued Tablets [ref: 4th quartile (3.2-9.6%)]			
1st quartile (0.10-0.79%)	1.04 (0.88 - 1.23)	0.89 (0.67 - 1.18)	0.96 (0.81 - 1.14)
2nd quartile (0.79-1.7%)	0.95 (0.80 - 1.14)	1.03 (0.77 - 1.38)	1.04 (0.88 - 1.25)
3rd quartile (1.7-3.2%)	1.00 (0.81 - 1.23)	1.12 (0.82 - 1.53)	1.00 (0.81 - 1.23)
Percent Patients with Broadband Access [ref: 4th quartile (93.5-96.2%)]			
1st quartile (35.7-86.5%)	0.95 (0.76 - 1.18)	0.62 (0.45 - 0.87)	1.06 (0.85 - 1.32)
2nd quartile (86.5-90.4%)	0.93 (0.78 - 1.12)	0.66 (0.48 - 0.89)	1.07 (0.89 - 1.29)
3rd quartile (90.4-93.5%)	0.94 (0.79 - 1.14)	0.77 (0.56 - 1.05)	1.06 (0.88 - 1.27)
**Mental Health Integration**			
Post-Pandemic Onset (versus Pre-)	16.74 (13.74 - 20.40)	9.89 (7.44 - 13.16)	0.060 (0.049 - 0.073)
Rural (versus Urban)	1.23 (0.83 - 1.80)	1.62 (0.87 - 3.00)	0.82 (0.56 - 1.20)
Post-Pandemic Onset by Rurality	0.49 (0.35 - 0.67)	0.34 (0.21 - 0.56)	2.06 (1.48 - 2.86)
Mean Charlson Comorbidity Index	1.07 (0.58 - 1.98)	0.43 (0.26 - 0.72)	0.93 (0.50 - 1.73)
Mean Number of Patients per Month	1.000 (1.000 - 1.000)	1.000 (1.000 - 1.000)	1.000 (1.000 - 1.000)
Percent Black Patients	1.005 (0.993 - 1.017)	0.992 (0.986 - 0.998)	0.995 (0.983 - 1.001)
Percent Hispanic Patients	0.997 (0.989 - 1.006)	1.001 (0.998 - 1.013)	1.003 (0.994 - 1.011)
Percent Patients with VA-issued Tablets [ref: 4th quartile (3.2-9.6%)]			
1st quartile (0.10-0.79%)	0.91 (0.61 - 1.36)	0.88 (0.64 - 1.22)	1.08 (0.74 - 1.63)
2nd quartile (0.79-1.7%)	1.00 (0.71 - 1.40)	0.86 (0.66 - 1.10)	1.00 (0.71 - 1.40)
3rd quartile (1.7-3.2%)	0.77 (0.54 - 1.10)	0.73 (0.57 - 0.94)	1.30 (0.91 - 1.87)
Percent Patients with Broadband Access (ref: 4th quartile (93.5-96.2%))			
1st quartile (35.7-86.4%)	0.93 (0.58 - 1.47)	0.63 (0.42 - 0.95)	1.08 (0.68 - 1.72)
2nd quartile (86.4-90.4%)	1.07 (0.78 - 1.47)	1.07 (0.80 - 1.43)	0.94 (0.68 - 1.28)
3rd quartile (90.4-93.5%)	1.10 (0.82 - 1.48)	0.80 (0.66 - 1.00)	0.91 (0.68 - 1.22)

[a]Telemedicine visits include telephone, video, and secure messaging.

Abbreviations: OR, odds ratio; CI, confidence interval.

© 2023 Leung LB et al. *JAMA Network Open.*

eTable 4. Incidence Rate Ratios and 95% CIs for Telemedicine, Video, and In-Person Visits in Primary Care and Mental Health Integration Services, Using Multivariable Negative Binomial Models

**Primary Care**	**IRR (95% CI)**	***P***
Post-Pandemic Onset (versus Pre-)	1.00 (0.99, 1.01)	0.751
Rural (versus Urban)	1.00 (0.98, 1.02)	0.942
Post-Pandemic Onset by Rurality	0.98 (0.96, 0.99)**	0.006
**Mental Health Integration**	**IRR (95% CI)**	***P***
Post-Pandemic Onset (versus Pre-)	1.01 (0.99, 1.02)	0.412
Rural (versus Urban)	0.99 (0.96,1.03)	0.629
Post-Pandemic Onset by Rurality	0.97 (0.939, 0.998)*	0.039

Abbreviations: IRR, incidence rate ratio.

© 2023 Leung LB et al. *JAMA Network Open.*

eTable 5. Predicted Number of Visits[a] for Primary Care and Mental Health Integration Specialties, by VA Healthcare System Rurality and Pandemic Onset

	Healthcare System Rurality	Predicted Number of Visits per Site (95% CI)
**Primary Care**		
Pre-Pandemic	Urban	162,220 (160,251 -164,189)
	Rural	162,109 (159,817 - 164,402)
Post-Pandemic Onset	Urban	161,929 (159,342 - 164,516)
	Rural	158,075 (155,111 - 161,039)
**Mental Health Integration**		
Pre-Pandemic	Urban	9,362 (9,172 - 9,553)
	Rural	9,280 (9,004 - 9,556)
Post-Pandemic Onset	Urban	9,426 (9,221 - 9,631)
	Rural	9,047 (8,816 - 9,279)

[a]Derived from multivariable negative binomial regression analysis with patient count per site as an offset.

© 2023 Leung LB et al. *JAMA Network Open.*
