# Supplementary material for: Rates of Primary Care and Integrated Mental Health Telemedicine Visits Between Rural and Urban Veterans Affairs Beneficiaries Before and After the Onset of the COVID-19 Pandemic
Source: JAMA Netw Open. 2023 Mar 7;6(3):e231864. doi: 10.1001/jamanetworkopen.2023.1864 (PMC9993180; doi:10.1001/jamanetworkopen.2023.1864)
Supplement: Supplement 2. — Data Sharing Statement [file jamanetwopen-e231864-s002.pdf]

Data Sharing Statement

Leung. Rates of Primary Care and Integrated Mental Health Telemedicine Visits Between Rural and Urban Veterans Affairs Beneficiaries Before and After the Onset of the COVID-19 Pandemic. *JAMA Netw Open*. Published March 07, 2023.
doi:10.1001/jamanetworkopen.2023.1864

Data
Data available: No

Additional Information
Explanation for why data not available: Under the Health Insurance Portability and Accountability Act (HIPAA), the dataset used in this study cannot be shared publicly because it contains patient-level Protected Health Information/Personally Identifiable Information (PHI/PII) from the Veterans Health Administration. To gain access to this data, interested researchers must complete credentialing to conduct VA research, as well as data use agreements with the Primary Care Analytics Team (pcat@va.gov) and other relevant VHA data owners.
